# Progranulin promoted the proliferation, metastasis, and suppressed apoptosis via JAK2-STAT3/4 signaling pathway in papillary thyroid carcinoma

**DOI:** 10.1186/s12935-023-03033-2

**Published:** 2023-09-02

**Authors:** Yanxu Dong, Hao Tan, Lidong Wang, Zhen Liu

**Affiliations:** Department of General Surgery, Shengjing Hospital of China Medical University, 36 Sanhao Street, Heping District, Shenyang, 110004 China

**Keywords:** Thyroid neoplasm, JAK2-STAT3/4 pathway, Progranulin, Invasion, Apoptosis, JSI-124

## Abstract

**Background:**

Progranulin (PGRN), a glycoprotein secreted by a wide range of epithelial cells and plays an important role in inflammatory mechanisms and tumor progression. In this study, the expression, and functions of PGRN in papillary thyroid carcinoma (PTC) was examined to explore the potential pathogenesis of PTC.

**Methods:**

Western blotting and qRT-PCR were used to detect the relationship between PGRN expression and clinicopathological characteristics of patients with PTC. PTC cell lines with PGRN overexpression and with PGRN knockdown were established to explore their effects on the biological behavior. Western blotting was used to detect the changes of relevant molecules and JAK2-STAT3/4 signaling pathway. Moreover, rescue experiments validated the involvement of the JAK2-STAT3/4 signaling pathway. And statistical analyses were analyzed using SPASS 21.0 and graph generation were performed using GraphPad Prism 8.0.

**Results:**

PGRN was overexpressed in PTC tissue and increased by 75% at mRNA level and 161% at relative protein level in the patients with lymph node metastasis compared to without lymph node metastasis. Besides, PGRN regulated and promoted PTC cell proliferation, migration, invasion, and inhibited cell apoptosis. With PGRN overexpressed, relevant molecules including the expression of BCL2/BAX, BCL2/BAD, CyclinD1, MMP2, vimentin and N-cadherin were increased, the expression level of E-cadherin was decreased, and the phosphorylation of JAK2 and STAT3/4 were increased. JAK inhibitor (JSI-124) rescued these changes of PTC cells induced by overexpressed PGRN.

**Conclusions:**

These findings revealed that PGRN promote the progression of PTC through the JAK2-STAT3/4 pathway, and PGRN could be served as a potential therapeutic target for PTC.

## Background

Thyroid carcinoma is one of the most general endocrine malignancies, its incidence has markedly risen worldwide over the past 40 years [1]. By 2030, it is predicted that thyroid carcinoma will be the second most general carcinoma in female and the ninth most common in male [2]. Papillary thyroid carcinoma (PTC) is the most prevalent type, counting for 84% in all types of thyroid cancer [3]. From 1994 to 2013, incidence-based mortality increased approximately 1.1% per year overall and 2.9% per year for advanced-stage papillary thyroid cancer [1]. The risk factors for thyroid cancer include ionizing radiation, family history of thyroid cancer [4] and some chromosomal alterations [5]. And other low risk factors include thyroid imaging with iodine 131、iodine deficiency、high thyroid stimulating hormone(TSH)、autoimmunity、thyroid nodule and obesity [6, 7]. Thyroid cancers traditionally present with a palpable thyroid nodule, and detection with palpation accounts for about 30–40% of thyroid diagnoses [8]. Nowadays some new diagnostic methods are being used such as neck ultrasonography and other new techniques which results in overdiagnosis [9]. Although most PTCs are relatively indolent with a 10-year survival rate of patients more than 90%, there is still a poor prognosis for the 10% of patients diagnosed with advanced stages [10]. According to the follow-up survey, the recurrence rate of PTC is as high as 25% [11]. Currently, the treatment of PTC is often multidisciplinary, surgery as the main treatment method and the first choice for PTC, is not satisfactory for patients with highly invasive or metastatic tumors [12]. Postoperative treatment includes radioactive iodine ablation [13] and TSH suppression [14]. Significant scientific advances in the molecular characteristic of thyroid cancer have improved the use of more targeted treatments for advanced cancers, for example vemurafenib and selumetinib [15]. Furthermore, some tumors are heterogeneous with more aggressive mutations and different clinical, pathological, and molecular features. These pathological subtypes are considered to have a moderate risk of recurrence, and the pressure and burden on the patients need to be paid more attention [16]. Therefore, intensive research on the occurrence of PTC and elucidation of the underlying molecular mechanisms which results in the progression of PTC are crucial for new techniques to diagnose and treat the disease.

Progranulin (PGRN), a protein encoded by the progranulin gene (*GRN*) which is located in human chromosome 17, as well as a secreted protein secreted by epithelial cells [17]. The intact PGRN sequence is rich in cysteines and consists of 7.5 repetitive sequences from N-terminus to C-terminus, including a half-granular domain and seven granulin domains (1G-2 F-3B-4 A-5 C-6D-7E) [18]. PGRN can be secreted by lots of epithelial tissues, expressed in various cell types. Specifically, PGRN is located in lysosome [19] and can regulate lysosomal function through acidification of lysosomes [20]. As a growth factor, it participates in the events of inflammation, wound healing regulation and cell growth [21]. Interestingly, studies have found that the expression level of PGRN increased in malignant tumors, and it took part in the proliferation, metastasis, and apoptosis of tumor cells. For example, the expression level of PGRN in different breast epithelial cells was strongly related to the degree of tumorigenicity, tumor grade, proliferation index, P53 expression and other clinicopathological characteristics [22], and high PGRN expression in breast cancer cells can enhance the proliferation and migration ability [23]. In ovarian cancer, PGRN was regulated and controlled by multiple signaling including cAMP, MAPK signaling pathway [24] and mTOR signaling pathway [25] to stimulate its secretion and promote cell proliferation and survival as well. What’s more, PGRN as a ligand could bind to Eph receptors in bladder cancer and help regulate tumor cell motility, invasion, unanchored growth, and tumor formation in vivo depend on MAPK signal [26]. Recent studies have demonstrated that elevated serum level of PGRN in PTC patients. In addition, evaluating the clinicopathological characteristics of PTC patients found that the proportion of patients with primary lesion size > 1 cm were 3%, 5%, 8%, 8% respectively, and the proportion of microscopic/gross extrathyroidal extension (ETE) were 8%/0%, 9%/1%, 11%/1%, 11%/2% respectively based on the PGRN level quartiles. And median serum PGRN level was significantly higher in patients with primary lesion size > 1 cm than in patients with papillary thyroid microcarcinoma [27]. It can be speculated that PGRN may participate in the invasion and metastasis of PTC, however, its expression, biological function and molecular mechanism in PTC remain unclear.

In the study, by detecting the expression level of PGRN in PTC, the association between the expression of PGRN and the clinicopathological features of PTC patients were investigated. We also investigated the impacts of PGRN transfection on the typical biological behaviors of PTC cells in vitro. These findings for further research on the potential mechanism of PGRN to participate in the occurrence and development of PTC provides a new theory. PGRN may be considered as a diagnostic indicator and one of therapeutic targets for PTC.

## Materials and methods

### Clinical samples

Seventy specimens of PTC tissues and adjacent normal tissues were collected from patients in the Shengjing Hospital of China Medical University. The patient did not have any other therapy before surgery, and normal tissues were collected at least 2 cm away from the tumor area in the same patients with PTC. Immediately, all collected tissues were saved in liquid nitrogen. The study obtained informed consent from patients or guardians and was approved by the ethics Committee of Shengjing Hospital, China Medical University (approval number 2014PS47K).

### Cell culture

Normal thyroid follicular epithelial cell line Nthy-Ori-3-1 (N3) and PTC cell line K1 were obtained from European Collection of Authenticated Cell Culture System (ECACC, 2019). PTC cell lines TPC1 and BCPAP were obtained from BeNa Culture Collection (BNCC, 2021) and authenticated via STR Genotyping. K1 was maintained in the medium: 22.5% MCD105 (Abcam, Cambridge, USA), 22.5% DMEM/F-12 (CORNING, Jiangsu, China), 45% DMEM (CORNING, Jiangsu, China), 10% fetal bovine serum (FBS; Cat# S711-001 S, LONSERA, Ciudad de la Costa, Uruguay), and 1% penicillin and streptomycin (Gibco, Gaithersburg, USA). Nthy-Ori-3-1 cells were maintained in the medium: 90% RPMI-1640 (CORNING, Jiangsu, China), 10% fetal bovine serum, and 1% penicillin and streptomycin. TPC1 and BCPAP cells were maintained in the medium: 90% DMEM (CORNING, Jiangsu, China), 10% fetal bovine serum, and 1% penicillin and streptomycin. They were cultured at 37 °C in a 5% CO_2_ atmosphere.

### Quantitative reverse-transcription polymerase chain reaction (qRT-PCR)

The total RNA extraction by using TRIzol reagent (Cat# 9108; Takara, Beijing, China) with reference to the instructions. Complementary cDNA was obtained with PrimeScrip RT kit (Cat# RR047A; Takara, Beijing, China), and amplified reaction was obtained with TB Green ® Premix Ex Taq TM II kit (Cat# RR820; Takara, Beijing, China). qRT-PCR analysis was performed for the detection of PGRN using ABI 7500 Fast Real-^Time P^CR System. *GRN*: forward primer 5’- ATCTTTACCGTCTCAGGGACTT-3’, reverse primer 5’- CCATCGACCATAACACAGGCAC-3’. The amplification conditions: 95 °C for 30 s, 95 °C for 5 s, and 60 °C for 30 s, for a total of 40 cycles. The relative expression level was detected by 2^−ΔΔCt^ and -ΔCt methods. GAPDH was selected as the reference gene for *GRN*.

### Western blotting

The total proteins were extracted with RIPA buffer at 4 °C for 30 min and the homogenates were centrifuged at 14,000 rpm at 4 °C for 45 min. The proteins went through SDS-PAGE and transferred to PVDF membranes. Subsequently, incubating the membrane in 5% skim milk at room temperature (22–25 °C) for 2 h, and then use primary antibody: anti-PGRN antibody (1:1000; Cat# A5124; ABclonal, Wuhan, China), anti-BAD (1:3000, Cat# 10435-1-AP; Proteintech Group, Inc., Chicago, United States), anti-BAX(1:8000; Cat# 50599-2-lg; Proteintech Group, Inc., Chicago, United States), anti-BCL2(1:2000, Cat# EPR175009; Abcam, Cambridge, England), anti-CyclinD1(1:1000; Cat# 55506T; Cell Signaling Technology, Inc., Boston, United States), anti-E-Cadherin(1:2000; Cat# 20874-1-AP; Proteintech Group, Inc., Chicago, United States), anti-N-Cadherin (1:2000; Cat# 22018-1-AP; Proteintech Group, Inc., Chicago, United States), anti-Vimentin (1:5000, Cat# 10366-1-AP; Proteintech Group, Inc., Chicago, United States), anti-MMP2 (1:1000; Cat# 10373-2-AP; Proteintech Group, Inc., Chicago, United States), anti-JAK2 (1:800; Cat# YM0385; ImmunoWay, Texas, United States), anti-p-JAK2 (1:800; Cat# YP0306; ImmunoWay; Texas, United States), anti-STAT3 (1:800; Cat# YT4443; ImmunoWay, Texas, United States), anti-p-STAT3 (1:800; Cat# YM3641; ImmunoWay, Texas, United States), anti-STAT4 (1:800; Cat# YN5464; ImmunoWay, Texas, United States), anti-p-STAT4 (1:800; Cat# YP0252; ImmunoWay, Texas, United States), anti-GAPDH (1:10,000, Cat# 10494-1-AP; Proteintech Group, Inc., Chicago, United States). After incubation at 4 °C overnight, secondary goat anti-rabbit or anti-mouse IgG (1:2,000; Zhongshan Jin Qiao, Beijing, China) antibody was used for 2 h at room temperature (22–25 °C) and detected by enhanced chemiluminescence kit (Beyotime Biotechnology). The integrated optical density of each band was measured with Image-Pro Plus 6.0 software. GAPDH was used as a control to calculate the target relative protein expression level.

### Immunohistochemistry

Embedded tissue specimens in paraffin, 10% formalin fixed, sections at 3 μm thick. The expression of PGRN was measured with TM SP (mouse/rabbit) IHC kit (Cat#KIT-9720; Maixin, China). Rabbit PGRN monoclonal antibody (Cat#A20806; ABclonal, Wuhan, China) at a 1:200 dilution was used to incubate the slides. Then use a 3,3′-diaminobenzidine (Cat# DAB-0031; MXB, Maixin, China) solution to stain the slides for 2 min, counterstained with hematoxylin, dehydrated, covered with coverslips, and analyzed by light microscopy. The negative control group was treated with PBS instead of the primary antibody. Brown and yellow staining can be observed in the cytoplasm and cytomembrane, which are positive staining.

### Transfection

Cell transfection was performed with two PTC cell lines (TPC1and BCPAP). Cells were planted into 6-well plates before transfection. si-RNA, si-RNA-NC (negative control) was introduced into cells using Lipo3000 (Invitgen, Carlsad, CA, USA) with reference to the instructions, as the time of the cell density reaching 60–70% confluence. PGRN siRNA was purchased from GenePharma (Shanghai, China). Specifically, *GRN* siRNA sequences: sense:5’-GCUUCCAAAGAUCAGGUAATT-3’; antisense: 5’-UUACCUGAUCUUUGGAAGCTT-3’. Negative control sequences: sense: 5’-UUCUCCGAACGUGUCACGUTT-3’; antisense: 5’-ACGUGACACGUUCGGAGAATT-3’. The final siRNA concentration is 100 nM. Then the cells were collected for qRT-PCR, western blotting, and other detection. qRT-PCR was used to verify the efficiency after the transfection experience as well. By using HANBIO lentiviral gene transfection (Shanghai, China), the *GRN* was introduced into TPC1 and BCPAP cells to establish a cell line with high PGRN expression and a corresponding negative control cell line. Stable expression cell lines were screened with 2 µg/ml puromycin. Then, add 10 µM JSI-124 (Cat# HY-N1405; MedChemExpress, New Jersey, United States) to the medium of PGRN overexpressing cell lines and culture for 48 h so that to extract the total protein.

### MTS and colony formation assay

Viability of cells was detected with the CellTiter 96® AQ_ueous_ One Solution Cell Proliferation Assay (Cat# G3580; Promega, USA). The transfected cells were resuspended, diluted to 2 × 10^4^ cells/ml, and planted into a 96-well plate. Then 20 µL of MTS solution was added to each well at the culturing time of 24, 48, 72, and 96 h. Incubated at 37℃ for 4 h, then the absorbance at 490 nm with a multi-mode microplate reader was detected. Proliferative ability of cells was tested by colony formation assay as well. 6-well plates were used to plant cells at a density of 800 cells/well and incubated for 2 weeks. Finally, fixed cells with 4% paraformaldehyde and stained cells with crystal violet. The number of colonies in different wells was calculated. All above experiments were repeated at least three times.

### Cell proliferation assay (EdU)

BeyoClickTMEdU-594 cell proliferation detection kit (Cat# C0078S; Beyotime Biotechnology) was used, the transfected cells were planted into 12-well plates. After the cells were cultured overnight and returned to a normal state, removing half of the culture medium, and adding an equal volume of 2× EdU working solution (the final concentration was 20 µM) for 2 h. After labeling, add 500 µl of fixative solution at room temperature for 15 min, then wash with washing solution for 3 times, 5 min each time. Permeabilization solution was used in each well for 15 min. Prepare Click Additive Solution according to the instructions, add 200 µl to each well at room temperature in the dark for 30 min. To detect the cell proliferation ratio, Hoechst 33,342 was used for cell nucleus staining, 500 µl of 1× Hoechst 33,342 solution was added to each well at room temperature in the dark for 10 min. Fluorescence detection then can be performed. The experiments were performed at least three times.

### Wound healing assay

Wound healing assay can detect the cell migration ability. Specifically, the transfected cells were inoculated into 6-well plates until the cell density approached 90%. Then, use a 200 µl pipette to scratch the surface of plates, the suspended cells were washed by PBS. After incubation for 24 h in the serum-free medium, taking photographs corresponding to each scratch at 0 and 24 h in the same field of view under the microscope. Calculate the cell migration rate: wound closure rate (%) = [(0 -24 h)/0 h scratch area] ×100. These experiments were performed at least three times.

### Transwell assay

Cell invasive capacity was examined by using Transwell chambers (8 μm; Costar, Corning, New York City, USA) containing Matrigel. Transfer 200 µL of medium containing 5 × 10^4^ cells into the upper chamber without serum, then add 600 µL culture medium containing 20% fetal bovine serum to the lower chamber. They are incubated at 37 °C and 5% CO_2_ for 36 h, then fixed in the upper chamber with 4% paraformaldehyde and stained with crystal violet. The number of cells migrating through the membrane could be observed. These experiments were also performed at least three times.

### Flow cytometric assays

Cell cycle: Cells on 6-well plates were digested by free EDTA trypsin, the suspended ones were collected, and centrifuged at 2000 rpm for 5 min. Remove the supernatant, fix the cells with 500 µl of pre-cooled 70% ethanol overnight at − 20 °C. Thereafter, 500 µl of 9:1 PI/RNaseA staining solution (KeyGen Biotech, Nanjing, China) was added to each sample after centrifugation at room temperature and incubated for 30 min. Finally, the proportion of cells undergoing G0/G1, S and G2/M phase was detected by flow cytometer (BD Biosciences, New York, USA).

Cell apoptosis: Annexin V-FITC/PI (Cat# KGA105-KGA108; KeyGen Biotech, Nanjing, China) and Annexin V-APC/7AAD (Cat# KGA1023-1026; KeyGen Biotech, Nanjing, China) apoptosis detection kits were used in PGRN knockdown group and PGRN overexpression group respectively. After centrifugation, removing supernatant and adding 500 µl of Binding Buffer to the samples in the knockdown group, 5 µl of Annexin V-FITC and 5 µl of PI or 5 µl of Annexin V-APC and 5 µl of 7-AAD staining solution were added in the dark and incubated for 15 min. Then, flow cytometry was used (BD Biosciences, New York, USA) to detect the apoptosis. These experiments were performed at least three times.

### Bioinformatics

GeneMANIA (https://www.genemania.org) was operated to visual *GRN* and its related molecular function and PPI network. Genes co-expressed with *GRN* in the cBioPortal database (TCGA-THCA 2014 dataset, accessed 16 May 2022) was performed to make a functional and pathway enrichment analysis by using David (DAVID Functional Annotation Bioinformatics Microarray Analysis (ncifcrf.gov)). Gene set enrichment analysis was operated with GSEA 3.0 software. Download C2.cp.kegg.v6.1.symbols.gmt dataset from the Molecular Signatures Database on the GSEA website (Home (gesa.org.au)). Expression profile data and attribute files were grouped into PGRN^high^ and PGRN^low^ by using the default weighting method enrichment analysis. Setting the random classification frequency to 1,000.

### Statistical analysis

Our results are showed as mean ± SEM deviation of three experiments. Using SPASS 21.0 (IBM Corporation, Armonl, NY, USA) to analyze statistics and GraphPad Prism 8.0 to display graph. t test was used for comparison of the expression of PGRN in PTC tissues and access the relationships between the expression results of PGRN and clinicopathological characteristics of PTC. Correlation between two variables was analyzed by Spearman correlation coefficient while comparison among multiple samples was analyzed by one-way analysis of variance (ANOVA). *P* < 0.05 was regarded statistically significant. ***, *P* < 0.001; **, *P* < 0.01; *, *P* < 0.05.

## Results

### Expression of PGRN in papillary thyroid carcinoma

**Fig. 1:**
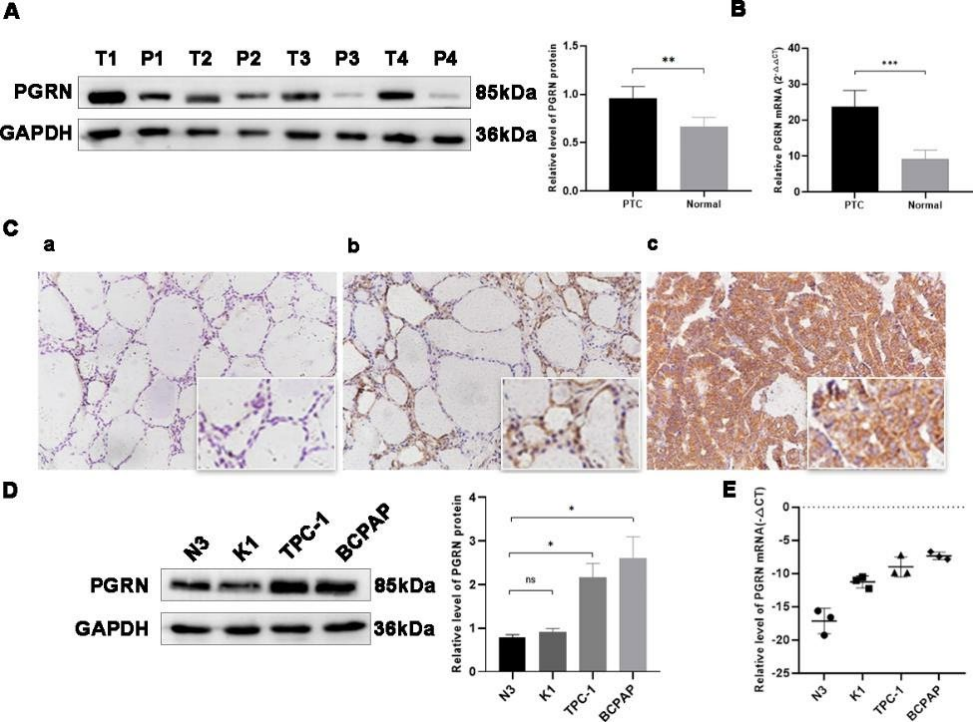
Expression of PGRN in papillary thyroid carcinoma (PTC) tissues and PTC cells. (**A**) Expression of PGRN in PTC and normal tissues adjacent to cancer was detected by using western blotting (n = 70 per group). Relative grayscale values of PGRN in PTC tissues and normal tissues adjacent to cancer. (**B**) Expression of PGRN in papillary thyroid carcinoma (PTC) tissues and normal tissues adjacent to cancer was detected by qRT-PCR (n = 70 per group). (**C**) Expression of PGRN in PTC samples (×200, low right ×400). (a) Negative expression of PGRN in normal tissues adjacent to cancer; (b) Positive expression of PGRN in normal tissues adjacent to cancer; (c) Positive expression of PGRN in PTC tissues; (**D**) Representative images and quantitation of the western blotting showed that the protein expression of PGRN in the PTC cell lines (K1、TPC1 and BCPAP) and normal thyroid epithelial cells (N3) (n = 3). GAPDH was used as an internal control. (**E**) The relative PGRN mRNA expression in PTC cell lines and normal thyroid epithelial cell (n = 3). Data are presented as mean ± SEM. *, *P* < 0.05; **, *P* < 0.01; ***, *P* < 0.001

**Fig. 2:**
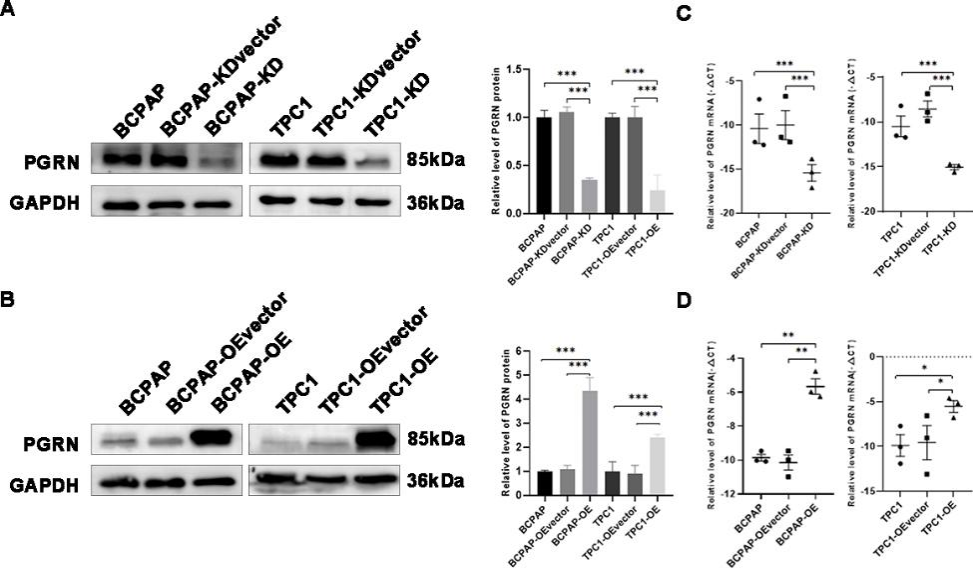
The prote in and mRNA expression of PGRN in the PGRN overexpression / knockdown groups. (**A, B**) Representative images and quantitation of the western blotting showed that the protein expression of PGRN in the PGRN overexpression/knockdown groups (n = 3). GAPDH was used as an internal control. (**C, D**) The relative PGRN mRNA expression in the PGRN overexpression/knockdown groups (n = 3). Data are presented as mean ± SEM. *, *P* < 0.05; **, *P* < 0.01; ***, *P* < 0.001

To explore the role of PGRN in thyroid carcinoma, the relative mRNA expression levels of PGRN in 70 PTC tissues and their paired paraneoplastic tissues were assessed. The qRT-PCR results showed that the higher relative mRNA expression level of PGRN in the PTC tissues than in adjacent normal tissues (23.827 ± 4.450 and 9.276 ± 2.412, respectively; *P* < 0.05) (Fig. 1B). Continuously, we detected the relative protein expression of PGRN by western blotting and immunohistochemical methods. The results of western blotting showed that the significantly higher protein expression level of PGRN in 70 PTC tissues than in adjacent normal tissues (0.958 ± 0.122 and 0.669 ± 0.091; *P* < 0.05) (Fig. 1A). Immunohistochemical result showed that PGRN was mainly expressed in the cytoplasm (Fig. 1C). Consistent with the clinical specimen data, PGRN overexpressed in PTC cell lines (TPC1 and BCPAP) than Nthy-ori3-1 cells. Furthermore, the expression of PGRN was highest in BCPAP cells (*P* < 0.05) (Fig. 1D, E). TPC1 and BCPAP cells were selected to construct high PGRN expression cell lines (TPC1-OE and BCPAP-OE) and PGRN knockdown cell lines (TPC1-KD and BCPAP-KD). Changes in the expression of PGRN were confirmed respectively by qRT-PCR and western blotting (Fig. 2).

A total of 70 PTC specimens were evaluated in the study. Table 1 shows the association between the expression level of PGRN and clinicopathologic parameters. According to the method of independent sample t test in medical statistics, compared to the group without lymph node metastasis, the PGRN mRNA expression level and PGRN protein expression level in the group with lymph node metastasis were significantly increased by 75% at mRNA expression level (2^−ΔΔCt^: 28.925 ± 5.867 vs. 11.081 ± 4.222; *P* < 0.05) and increased by 161% at relative protein expression level (1.127 ± 0.143 vs. 0.642 ± 0.174; *P* < 0.05). And other clinicopathological parameters such as age, gender, multifocality, extracapsular infiltration, and tumor size were not related to the expression level of PGRN (*P* > 0.05). The data suggested that PGRN can affect the malignancy of PTC, thereby promoting lymph node metastasis.

**Table 1:** Relationship between PGRN and the clinical pathological features in papillary thyroid carcinoma

Clinical features	n	PGRN protein	P	PGRN mRNA	P
Gender Male Female	12 58	1.178 ± 0.199 0.949 ± 0.134	0.076	24.639 ± 11.984 23.659 ± 4.826	0.887
Age ≥ 55 < 55	16 54	0.992 ± 0.173 0.987 ± 0.143	0.419	28.380 ± 9.406 22.477 ± 5.084	0.227
Tumor size ≤ 2 > 2	45 25	0.887 ± 0.146 1.172 ± 0.192	0.228	23.114 ± 5.574 25.109 ± 4.222	0.089
Lymph node metastasis Yes No	50 20	1.127 ± 0.143 0.642 ± 0.174	0.021	28.925 ± 5.867 11.081 ± 4.222	0.014
Mutiplicity Yes No	32 38	0.936 ± 0.200 1.032 ± 0.135	0.197	26.552 ± 7.075 21.532 ± 5.692	0.568
Bilateral Yes No	16 54	1.053 ± 0.140 0.772 ± 0.189	0.259	24.735 ± 5.416 20.761 ± 6.922	0.685
Extra thyroid invasion Yes No			0.045		0.794
	4 66	1.839 ± 0.560 0.937 ± 0.117		18.764 ± 12.613 24.134 ± 4.672	

### PGRN affects the proliferative capacity of PTC

**Fig. 3:**
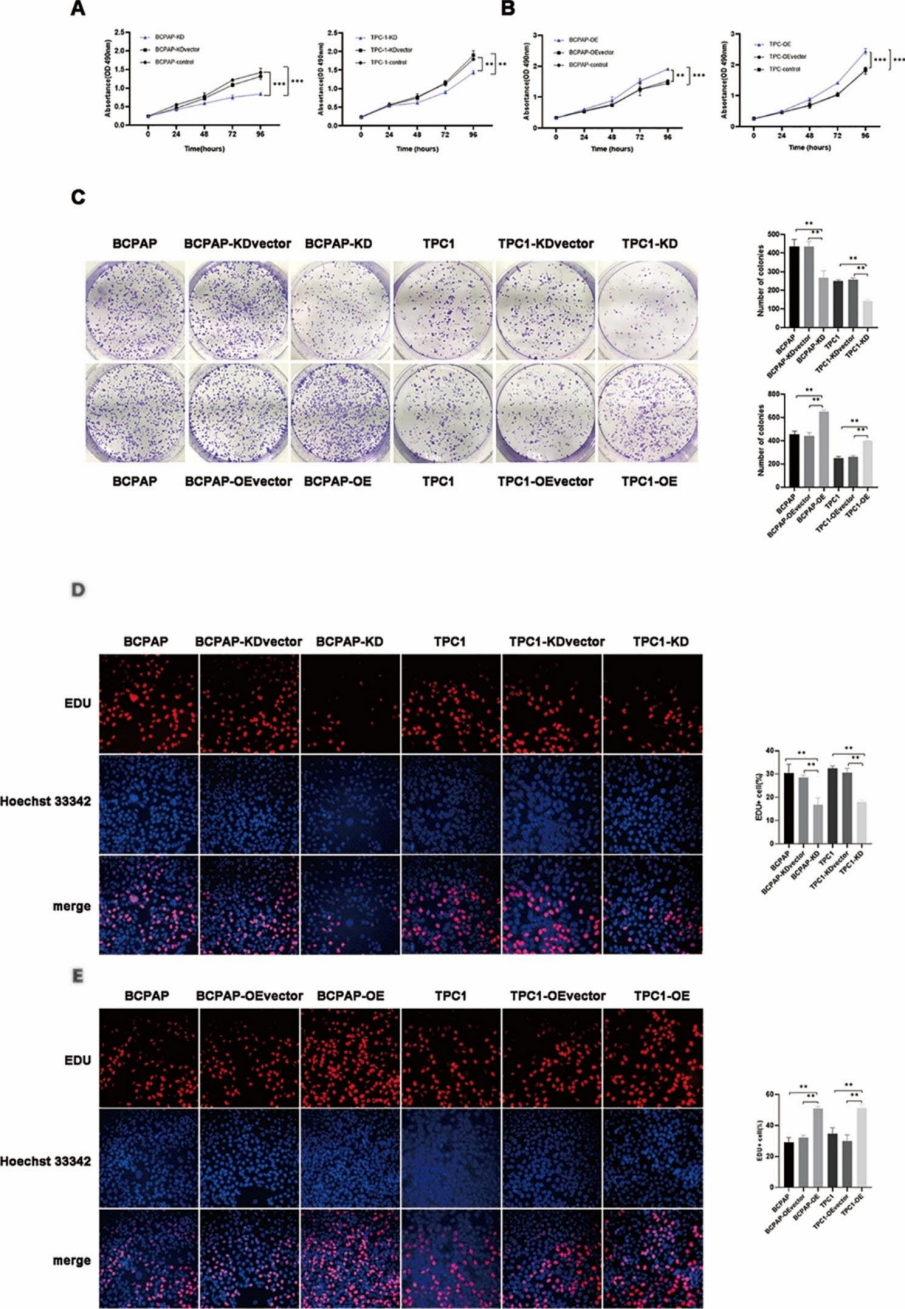
The influences of PGRN on proliferation in PTC cells. (**A, B**) Knockdown of PGRN inhibited cell proliferation of ovarian cancer cells in MTS assay (n = 3). Overexpression of PGRN promoted cell proliferation of ovarian cancer cells in MTS assay (n = 3). (**C**) Colony formation assay present the proliferation of knockdown/overexpression of PGRN (n = 3). (**D, E**) EDU assay presented the proliferation of knockdown/overexpression of PGRN (n = 3). Data are presented as mean ± SEM. *, *P* < 0.05; **, *P* < 0.01; ***, *P* < 0.001

TPC1 and BCPAP cells were chosen to construct PGRN overexpression cell lines (BCPAP-OE and TPC1-OE) and PGRN knockdown cell lines (BCPAP-KD and TPC1-KD). Changes of PGRN expression after transfection experience were verified by qRT-PCR and Western blotting (Fig. 2**)**. The proliferative capacity of these cells was assessed by calculating the rate of cell proliferation after PGRN overexpression/knockdown. MTS assay showed that PGRN knockdown could reduce the cell viability significantly (*P* < 0.05) (Fig. 3A). Similarly, the cell viability of PGRN overexpression was significantly higher than that in the control group (*P* < 0.05) (Fig. 3B). The colony formation assay showed that the colonies in the PGRN knockdown group was remarkably decreased compared to the control group (*P* < 0.05) (Fig. 3C), and the colonies was also remarkably increased after PGRN overexpression (*P* < 0.05) (Fig. 3C). Additionally, EDU test turns out the same conclusion. The percentage of EDU-positive cells in PGRN knockdown group significantly decreased (*P* < 0.05) (Fig. 3D). In contrast, the percentage of EDU-positive cells in PGRN overexpression group significantly increased (*P* < 0.05) (Fig. 3E). These results show that high PGRN expression promotes the proliferation of PTC cells.

### PGRN overexpression inhibits apoptosis of PTC cells

**Fig. 4:**
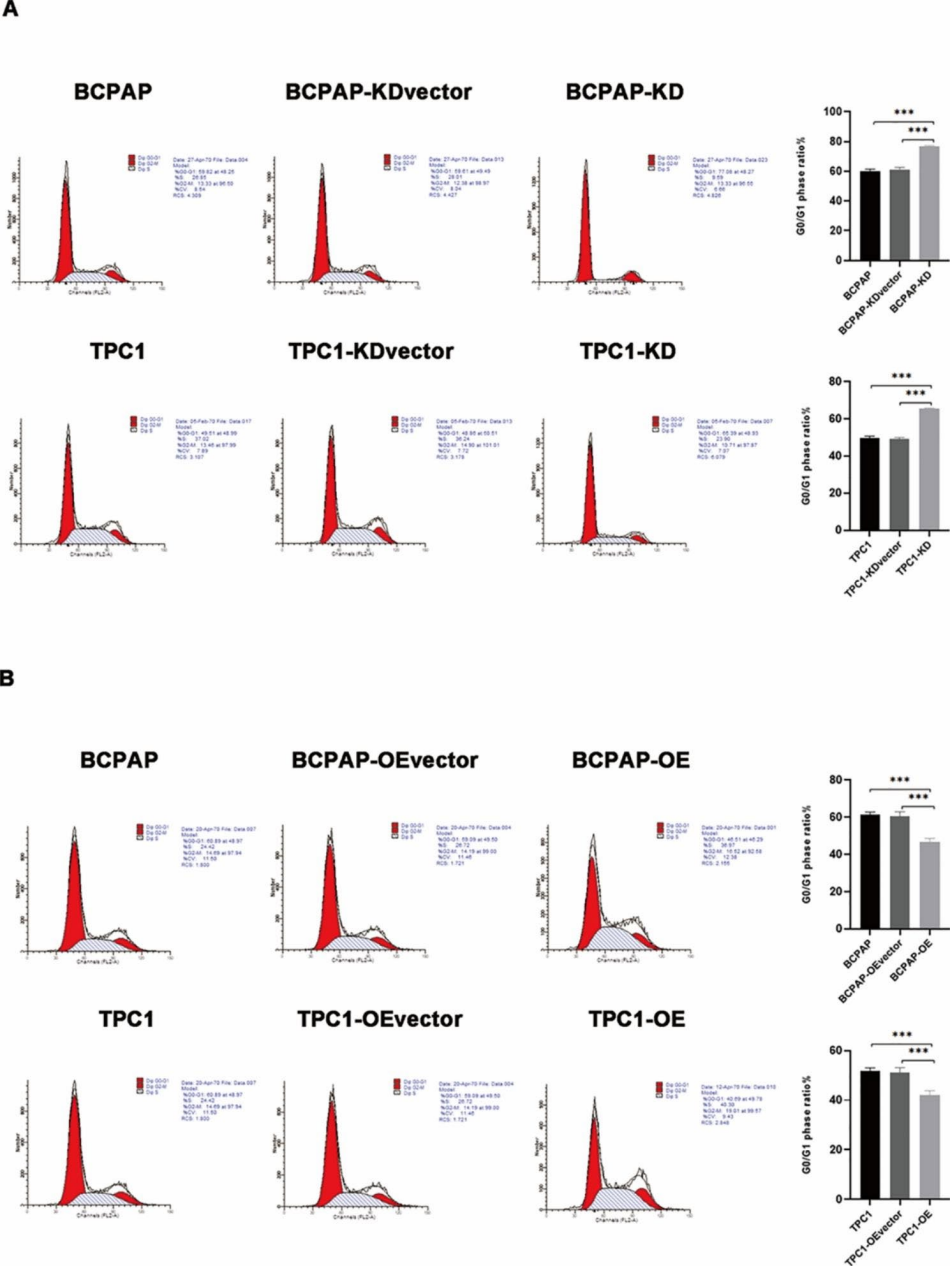
Flow cytometry analysis of the influences of PGRN on cell cycle in PTC cells by PI/RNaseA staining. (**A**) Knockdown of PGRN induced PTC cells arrest in the G0/G1 phase (n = 6). (**B**) Overexpression of PGRN promoted PTC cell cycle passed into S and G2/M phase (n = 6). Data are presented as mean ± SEM. *, *P* < 0.05; **, *P* < 0.01; ***, *P* < 0.001

**Fig. 5:**
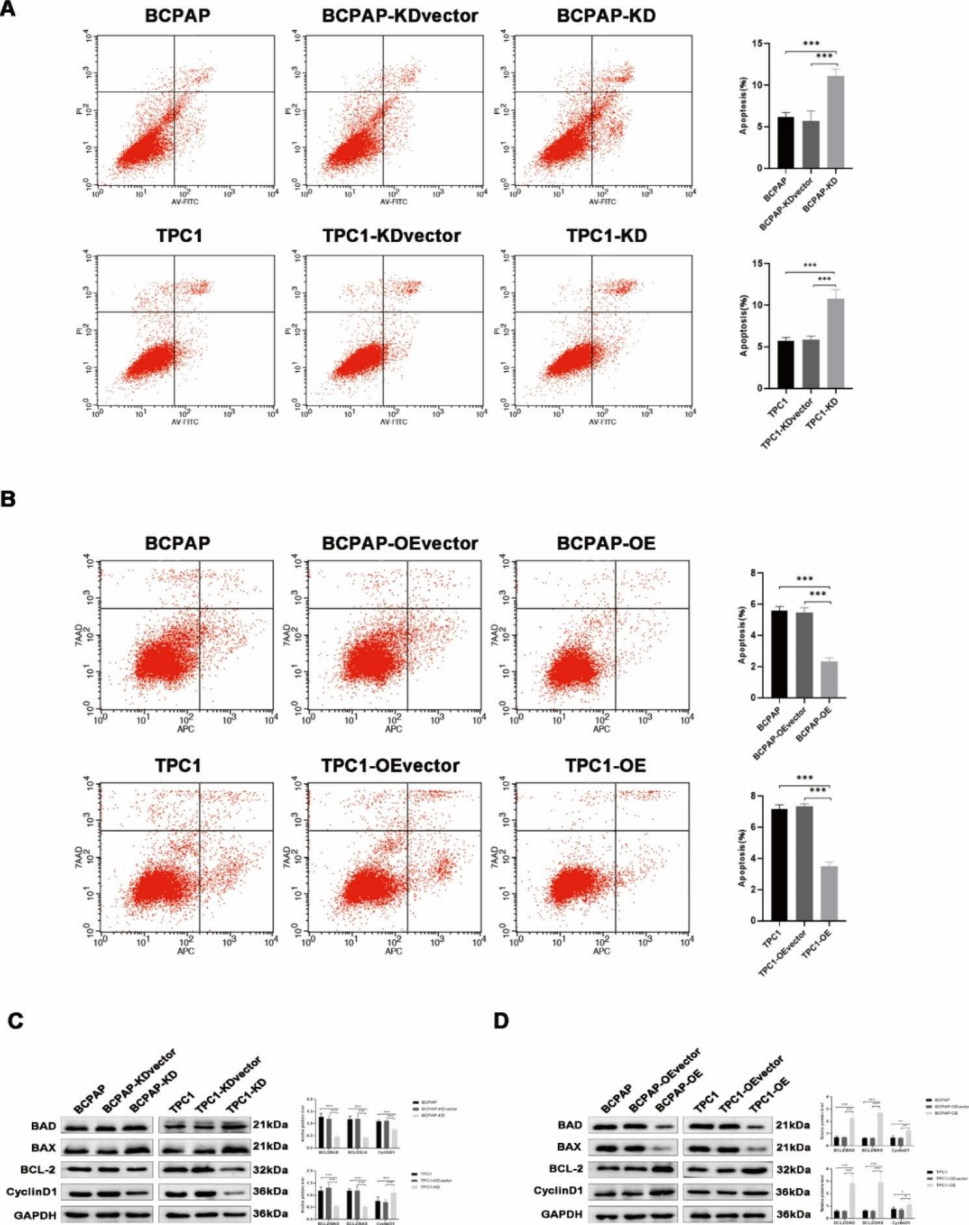
Flow cytometry analysis of the influences of PGRN on apoptosis in PTC cells by Annexin V-FITC-PI or Annexin V-APC/7AAD staining. (**A**) Knockdown of PGRN promoted the apoptosis of PTC cells (n = 6). (**B**) Overexpression of PGRN inhibited the apoptosis of PTC cells (n = 6). (**C, D**) Representative images and quantitation of the western blotting showed that the protein expression of BAD、BCL2、BAX and CyclinD1 in the PGRN knockdown/overexpression groups (n = 3). GAPDH was used as an internal control. Data are presented as mean ± SEM. *, *P* < 0.05; **, *P* < 0.01; ***, *P* < 0.001

Flow cytometry analysis suggested that PGRN overexpression promoted the transition of cell cycle from G1 phase to S phase (*P* < 0.05) (Fig. 4B), and the apoptosis rate significantly reduced (*P* < 0.05) (Fig. 5B). Conversely, PGRN knockdown resulted in cells arrested in G0/G1phase (*P* < 0.05) (Fig. 4A), and apoptosis rate of cancer cells significantly increased as well (*P* < 0.05) (Fig. 5A). To verify this effect, mitochondrial apoptosis-related markers were detected by western blotting. With the PGRN overexpressed, the expression level of BCL2 and CyclinD1 increased, but the expression level of BAX and BAD decreased (Fig. 5D). Conversely, after PGRN knockdown, the expression level of BCL2 and CyclinD1 decreased, and the expressions level of BAX and BAD increased (Fig. 5C), which was consistent with the results of flow cytometry. These results suggest that PGRN overexpression promotes the transition of PTC cells from G1 phase to S phase and inhibits PTC cell apoptosis.

### PGRN promotes PTC cell invasion and migration

**Fig. 6:**
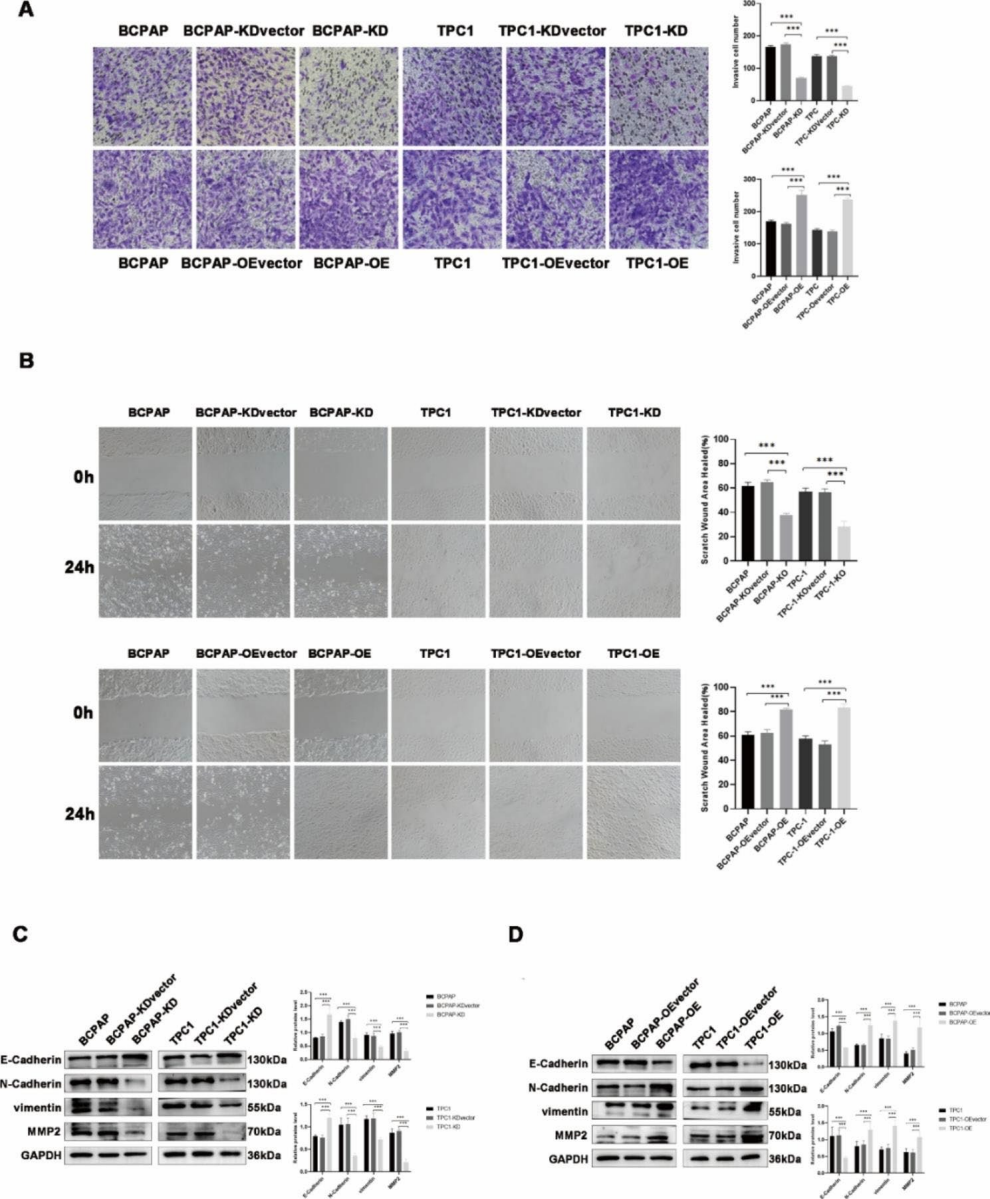
PGRN promoted invasion and migration in ovarian cancer cells. (**A**) Knockdown of PGRN suppressed PTC cells invasion, overexpression of PGRN promoted PTC cells invasion (n = 9;×200). (**B**) Knockdown of PGRN inhibited PTC cells migration, overexpression of PGRN promoted PTC cells migration (n = 9;×200, Scale Bar = 100 μm). (**C, D**) Representative images and quantitation of the western blotting showed that the protein expression of E-Cadherin, N-Cadherin, vimentin and MMP2 in the PGRN knockdown/overexpression groups (n = 3). GAPDH was used as an internal control. Data are presented as mean ± SEM. *, *P* < 0.05; **, *P* < 0.01; ***, *P* < 0.001

The influences of PGRN overexpression/knockdown on cell invasion and migration were next evaluated in the study. PGRN knockdown reduced the invasion and attack of cells (*P* < 0.05) (Fig. 6A), while PGRN overexpression significantly enhance the invasion and attack of cells (*P* < 0.05) (Fig. 6A). After PGRN gene knockdown, the wound healing rate of the cell was lower significantly, and the cell migration was reduced compared to the control group (*P* < 0.05) (Fig. 6B). After PGRN overexpression, the wound healing rate of the cell was higher, and the cell migration was enhanced compared to the control group (*P* < 0.05) (Fig. 6B). With PGRN knockdown, MMP2, vimentin, and N-cadherin were down-regulated, while E-cadherin was up-regulated (*P* < 0.05) (Fig. 6C). The contrary result was obtained with PGRN overexpression (*P* < 0.05) (Fig. 6D). These results further confirm that PGRN promotes PTC cell migration, invasion and EMT.

### PGRN activates the JAK2-STAT3/4 signaling pathway

**Fig. 7:**
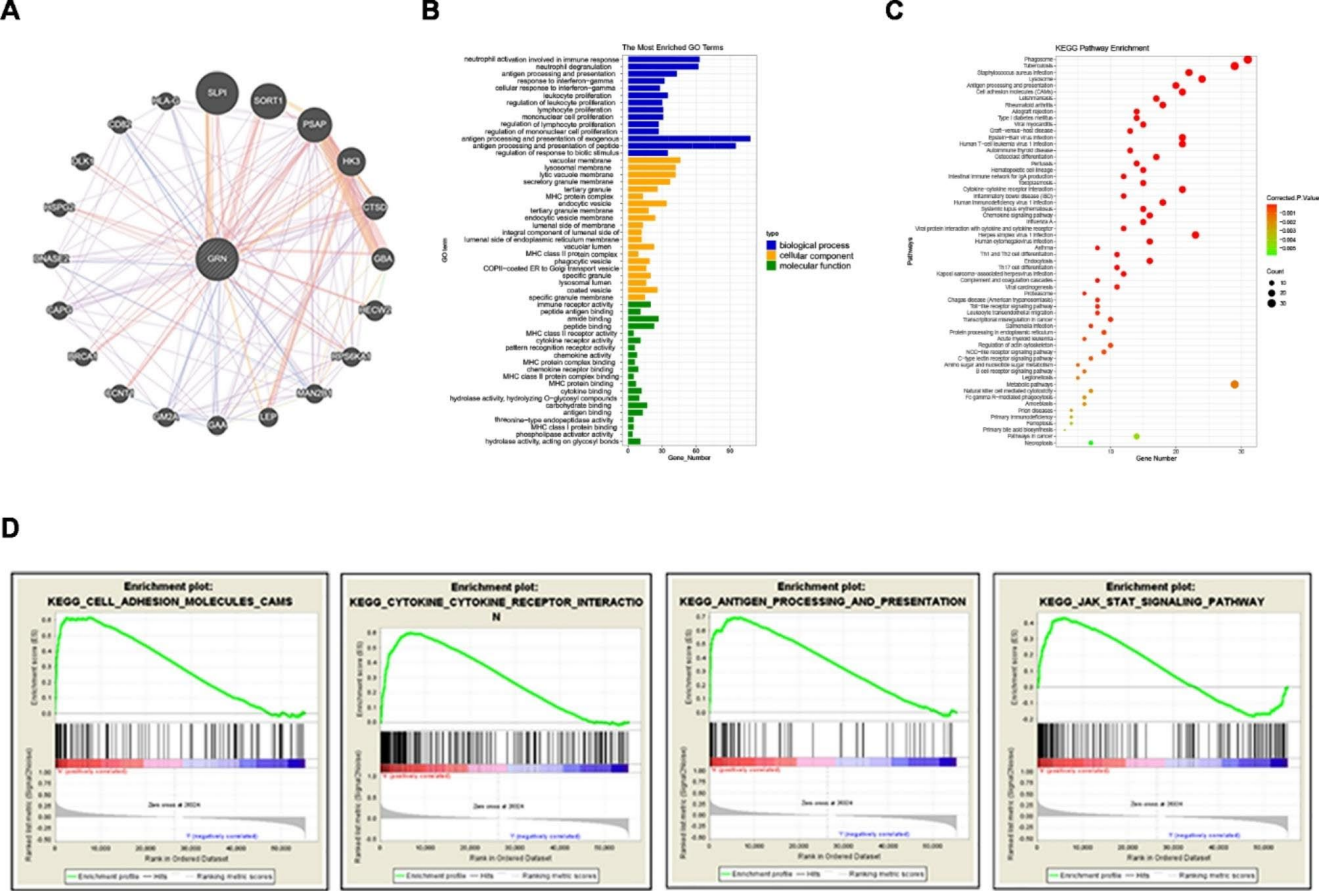
Gene interactions and enrichment analysis of PGRN. (**A**) Gene interaction network of the top 20 genes associated with PGRN in GeneMANIA. (**B**) Gene ontology enrichment analysis by genes co-expressed with PGRN. (**C**) Kyoto Encyclopedia of Genes and Genomes enrichment analysis by genes co-expressed with PGRN. (**D**) Gene set enrichment analysis showing PGRN in the published thyroid cancer dataset (The Cancer Genome Atlas database)

**Fig. 8:**
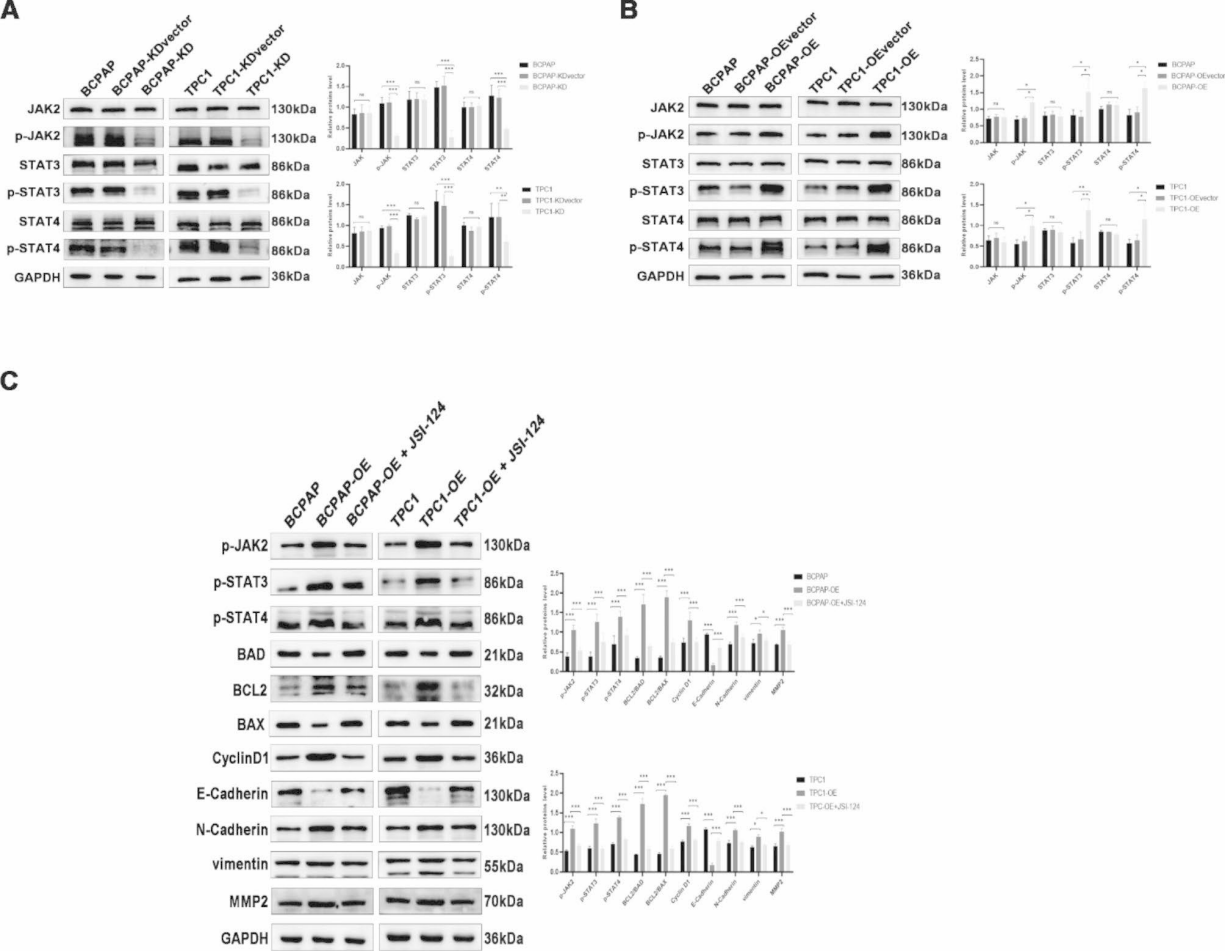
PGRN activated the JAK/STAT signaling pathway. (**A, B**) Representative images and quantitation of the western blotting showed that the protein expression of JAK2, p-JAK2, STAT3, p-STAT3, STAT4 and p-STAT4 in the PGRN overexpression/knockdown groups (n = 3). GAPDH was used as an internal control. Data are presented as mean ± SEM. *, *P* < 0.05; **, *P* < 0.01; ***, *P* < 0.001. (**C**) Representative images and quantitation of the western blotting showed that the protein expression of JAK2, p-JAK2, p-STAT3, p-STAT4, BAD、BCL2、BAX、CyclinD1、E-Cadherin、N-Cadherin、vimentin and MMP2 in the PGRN overexpression group with JAK inhibitor (JSI-124) (n = 3). GAPDH was used as an internal control. Data are presented as mean ± SEM. *, *P* < 0.05; **, *P* < 0.01; ***, *P* < 0.001

GeneMANIA website analysis of the top 20 associated genes of *GRN* to build a protein interaction network. Four most relevant genes including secretory leucocyte protease inhibitor (*SLPI*), sortilin1 (*SORT1*), prosaposin (*PSAP*), and cathepsin (*CTSD*) were physically interacted with *GRN* (Fig. 7A). Using the DAVID database and the “ggplot2” R package to understand the biological functions and pathways related with *GRN*, we performed functional and pathway enrichment analysis of genes who were screened co-expressing with *GRN* in the cBioPortal library. Gene Ontology enrichment analysis results showed that *GRN* co-expressed genes participated in various of processes, biological process including antigen processing and presentation of exogenous and peptide, neutrophil activation involved in immune response. They also referred to the cellular components containing vesicular and lysosomal membranes, and molecular functions such as amino and peptide binding, and immune receptor activity as well (Fig. 7B). What’s more, the enrichment analysis of *GRN*-related genes in the KEGG (Kyoto Encyclopedia of Genes and Genomes) pathways revealed that *GRN* was closely related to signaling pathways in cancer. Meanwhile, antigen processing and presentation, autoimmune thyroid disease, cytokine and cytokine receptor interaction, chemokine signaling pathway, which are associated with tumor immunity, were also enriched in *GRN* co-expressed genes (Fig. 7C). In addition, single-gene enrichment analysis was performed depending on published thyroid cancer dataset (TCGA-THCA). According to FDR q value, high PGRN expression were closely related with cell adhesion (FDR q value = 0.019), cytokine-receptor association (FDR q value = 0.005), regulation of antigen presentation (FDR q value = 0.004), and the JAK-STAT pathway (FDR q value = 0.130) (Fig. 7D). Although its FDR q value related to JAK-STAT signaling pathway was 0.130, the impact of PGRN on JAK-STAT signaling pathway was verified by western blotting experiments. With PGRN knockdown, the expression of p-JAK2, p-STAT3 and p-STAT4 decreased significantly (*P* < 0.05) (Fig. 8A). Similarly, with the overexpression of PGRN, the expression level of p-JAK2, p-STAT3 and p-STAT4 increased (*P* < 0.05) (Fig. 8B). However, the expression levels of JAK2, STAT3 and STAT4 almost have no change in both situations (*P* > 0.05) (Fig. 8A, B). This confirms that PGRN activates the JAK2-STAT3/4 signaling pathway.

To further explore the role of JAK-STAT signaling pathway in apoptosis and migration induced by PGRN, JSI-124 (JAK2 inhibitor) was used to inhibit the activation of JAK2, STAT3 and STAT4 in BCPAP-OE and TPC1-OE cells so that to evaluate whether JSI-124 can rescue the impact of PGRN on EMT and apoptosis. The results showed that JSI-124 significantly rescued the apoptosis and invasion of PGRN overexpression cells, up-regulate BAX and BAD, down-regulate the protein levels of Cyclin D1、BCL2. In addition, E-cadherin was up-regulated and N-cadherin, vimentin and MMP2 protein levels were down-regulated (Fig. 8C). These findings suggest that JAK inhibitor partially rescues the changes of apoptosis and migration of PTC cells induced by PGRN. In conclusion, PGRN inhibits apoptosis and promotes EMT through JAK2-STAT3/4 signaling pathway.

## Discussion

The incidence of thyroid carcinoma is increasing annually, and it becomes the most common endocrine tumor. PTC is the major subtype of thyroid carcinoma, accounting for 85% of all types of thyroid carcinomas [28]. Although PTC has a good prognosis, the therapeutic effect of patients with recurrent and advanced metastases is still limited [29]. For recurrent and metastatic cases, surgery should be performed and followed by iodine therapy and TSH suppression therapy. Besides, ablation therapy can be considered if surgery is not possible [30]. However, once a differentiated thyroid cancer was no longer responsive to radioiodine therapy, there is no option for active treatment. Currently, the US Food and Drug Administration (FDA) has approved target therapies for these advanced thyroid cancers, such as sorafenib [31], lenvatinib [32], cabozantinib [33]. Therefore, it is essential to explore the mechanism of PTC so that to help early screen, prevent, and treat this disease.

Progranulin (PGRN) is a secreted protein, also known as acroprotein, granular/epithelial precursor (GEP), proepithelial protein, PC cell-derived growth factor (PCDGF) and 88KDa protein, named for its discovery in a wide range of epithelial tissues [34]. Recently, more and more evidence has shown that PGRN made contribution to the occurrence and development of various tumors, stimulated the proliferation, metastasis, angiogenesis, and anticancer drug resistance of carcinoma cells. When the expression of PGRN was inhibited with shRNA in the colorectal cancer SW1116 cell line, the expression of proliferation-associated protein Ki-67 decreased as well [35]. In ovarian cancer and breast cancer, PGRN increased the invasion of tumor cells by upregulating MMP-2 and MMP-9 proteins [36]. Further, PGRN could be secreted from different cells and bind to receptor proteins to produce significant biological effects. Sortilin as the receptor was necessary for PGRN to induce breast cancer stem cell-like proliferation. It assisted PGRN to promote the function of cancer stem cells in vivo, promoted lung cancer metastasis and local skin infiltration in xenograft models [37]. Particularly, TNFR was found likely to act as a direct receptor of PGRN in colorectal cancer, thereby activating the AKT pathway to affect cell proliferation and migration [38]. The latest study found that the serum PGRN level was elevated in PTC patients, and their levels were significantly correlated with the proportion of patients with primary lesions > 1 cm and the proportion of microscopic and macroscopic extrathyroidal extension (ETE) [27]. In conclusion, PGRN plays an essential role in the development of various malignant tumors. This study aims to explore its character and internal mechanism in PTC.

Here, we discussed the character and regulatory mechanism of PGRN in PTC. The results suggested that PGRN was overexpressed at both mRNA and protein levels in PTC tissues, that is a new discovery in the field of PTC. PGRN expression level was significantly correlated with cervical lymph node metastasis in PTC (*P* < 0.05). Therefore, we could speculate that the expression of PGRN was an important indicator for evaluating PTC invasion. Li et al. [39] also found that PGRN overexpressed in esophageal carcinoma, and its high expression was positively correlated with lymph node metastasis. Importantly, the expression level of PGRN was significantly higher in PTC cells than that in normal thyroid follicular epithelial cells, and the highest expression level was in BCPAP cells derived from poorly differentiated [40], which indicated that the expression level of PGRN may be related to the degree of tumor differentiation, meanwhile involved in the malignant regulation of tumor, so that reflecting the malignant degree of tumor.

Tumor was a population of cells that transformed cells continue to proliferate, and typical characteristics of malignant tumors are as follows: cell proliferative ability increased, cell apoptotic ability decreased. When the oncogenes activated or the tumor suppressor genes inhibited, the cells will undergo malignant transformation, uncontrolled proliferation, and blocked apoptosis. In present study, we found that PGRN knockdown promoted apoptosis while arresting PTC cells in G0/G1 phase and inhibited PTC cell proliferation. In contrast, overexpression of PGRN promoted the transition of PTC cells from G0/G1 phase to S phase and promoted cell proliferation, while the apoptotic ability of PTC cells was inhibited. It was suggested that PGRN regulated cell cycle and DNA replication to promote the proliferation of PTC. The imbalanced state of cell proliferation and apoptosis processes affected tumor progression [41]. Furthermore, BCL2 family are the important molecules that regulate the intrinsic mitochondrial pathway of apoptosis, including the apoptosis inhibitor BCL-2, the apoptosis promoter BAX and the regulatory protein BAD [42]. With PGRN overexpression, the expression level of BCL2 and CyclinD1 increased and the expression level of BAX and BAD decreased. It indicated that PGRN affected the apoptosis of PTC cells by regulating the intrinsic mitochondrial pathway through BCL2 family members. Wang et al. [43] found elevated BAX expression and decreased BCL2 expression in the spinal cord tissue of GRN-/- mice, leading to uncontrolled apoptosis, which was consistent with our findings. In addition, by verifying the apoptosis-related markers of PARP and caspase-3, it was also confirmed that the expression of PGRN in lung tissue inhibited smoking-induced apoptosis of lung epithelial tissue [44]. In conclusion, PGRN could inhibit the apoptosis of PTC cells and promote tumor progression. Besides, the study also found that PGRN overexpression promoted the progression of EMT in PTC. EMT was an important progression in cell morphology and motility, which was generally regarded as the key mechanism of cancer cell migration and invasion [45, 46]. To explore the mechanism by which PGRN promoted PTC cell invasion, we examined the changes of EMT-related proteins after PGRN knockdown and overexpression, E-cadherin, N-cadherin, vimentin, and MMP2. E-cadherin was up-regulated, while contrary results showed in the PGRN knockdown group, suggesting that PGRN was involved in regulating EMT in PTC cells. Similarly, overexpression of PGRN in ovarian cancer also accompanied with increased vimentin expression and decreased E-cadherin expression, resulting in cell migration and invasion [47]. However, Monami et al. found that PGRN required not only the activation of focal adhesion kinase (FAK) by phosphorylation of paxlin and ERK, but also the activation of FAK with F-actin to promote migration and invasion in bladder cancer cells [48]. It suggested that PGRN plays an important role in promoting the EMT of PTC.

Furthermore, we performed pathway enrichment analysis in the GSEA database to know about the impact of PGRN on malignant biological behavior of PTC and found that PGRN was involved to the JAK-STAT signaling. It was well known that abnormal activation of the JAK-STAT pathway strongly made attribution to the occurrence, development, drug resistance and poor prognosis of various tumors, including lung cancer, colorectal cancer, breast cancer and PTC [49–52]. And the study provided undeniable evidence that structurally activated STAT signaling pathway, particularly STAT3, played an important role in cancer progression by preventing apoptosis. STAT3 were involved in tumorigenesis by regulating several genes, including promoting cell cycle progression, and inhibiting apoptosis pathways [53, 54]. The downstream JAK-STAT pathway also promoted the activation of PI3K and MAPK pathways, which were regarded as the most central cascade reactions in the process of thyroid carcinogenesis, reflecting a complex and interconnected network of molecules and pathways. Therefore, its mechanism of action in thyroid cancer is worth to explore. The study of JAK-STAT signaling pathway in thyroid cancer was less and existed a great controversy. Previous studies had found that abnormal activation of STAT3 in human thyroid cancer specimens, and its overexpression had a strong correlation with the pathogenesis of thyroid cancer [55, 56]. However, the opposite conclusion had been obtained by other researchers that STAT3 might be a negative regulator of thyroid tumor growth, so the JAK-STAT3 pathway was important for inhibiting the procession of thyroid cancer rather than promoting it [57, 58]. Under these research backgrounds, our study continually found that the JAK-STAT signaling pathway associated proteins were indeed dysregulated after PGRN overexpression and knockdown in PTC cells. We confirmed that the expression level of PGRN affected the phosphorylation of JAK2 and STAT3, and the activation of STAT4 was also affected at the same time, resulting in increased or decreased expression of BCL-2, BAX, BAD and CyclinD1 to regulate cell apoptosis. What’s more, EMT associated protein could be regulated as a result. This hypothesis was consistent with literature data that PGRN was elevated in breast and colorectal cancer tissues and positively correlates with STAT3 activation [59, 60]. In addition, during the treatment of PTC with BRAF inhibitor (PLX4032), it was also found that STAT3 was up-regulated resulting in cell resistance [61]. In contrast, Khan et al. found that curcumin synergistically enhanced the anticancer activity of cisplatin in PTC and CSC-like cells by activating JAK2 and STAT3 [62]. Furthermore, JSI-124 was then used to inhibit activation of JAK2 and STAT3 in BCPAP-OE and TPC1-OE cells. JSI-124, also known as cucurbitacin I, a JAK inhibitor with high selectivity for JAK2/STAT3 which would not influence other pathways including Akt, extracellular signal-regulated kinase 1/2, and c-Jun NH2-terminal kinase [63]. It has been widely confirmed to inhibit JAK-STAT signaling pathway [64, 65]. Finally, our results proved that the changes of EMT-related proteins and apoptosis-related proteins induced by PGRN overexpression were rescued after inhibiting JAK-STAT signaling pathway. We could conclude that PGRN regulated the expression of BAX、BAD、BCL2、cyclinD1 and EMT associated protein so that it inhibited the apoptosis and promoted invasion through activating JAK2-STAT3/4 signaling in PTC. However, the expression levels of EMT and apoptosis related proteins did not completely offset the effect of PGRN overexpression and returned to normal expression levels in PTC cells by using JAK inhibitor (JSI-124). In addition, the mechanism was not limited to JAK-STAT signaling pathway. More branches of the pathway still need to be explored in the future. The types of extracellular ligands that could activate the JAK-STAT pathway include growth factors, interferons, interleukins, and hormones. Consequently, PGRN as a secreted protein, we could speculate that it may as an inflammatory factor activate the JAK-STAT signaling pathway by competing with other inflammatory factors to bind with the corresponding receptors. The Eph receptor has been found to be a functional signaling receptor for PGRN, which provides a new way to understand the potential role of PGRN in cancer progression [66]. PGRN was found to replace the typical ligand of EphA2-A1 in bladder cancer, phosphorylation of EphA2 Ser897 induced by PGRN binding to EphA2 might induce crosstalk between other receptor tyrosine kinases, including other Eph receptors and epidermal growth factor receptor (EGFR) [67]. This crosstalk enhanced the activation of MAPK and AKT, which played an important role in tumor progression. As we know, activation of STAT3 directly induced the expression of BCL-2 family members [53]. Furthermore, JAK2/STAT3 activation enhanced metastasis via induction of EMT by the upregulation of EMT-inducing transcription factors (EMT-TFs, Snail, Zeb1, JUNB, and Twist-1) and increased cell motility via focal adhesion kinase (FAK) activation [68]. In colorectal cancer, EMT and stemness maintenance were controlled simultaneously by inducing the IGF/STAT3/NANOG/Slug axis [69]. STAT3 .as a transcriptional activator also could bind to the promoter of the target gene in a tyrosine phosphorylation-dependent manner. And Hendrayani et al. found IL-6 and TGF-β1 promoted the binding of STAT3 to the AUF-1 promoter, reducing the levels of p16, p21 and p53 by paracrine means, leading to the activation of breast stromal fibroblasts. In turn, elevated AUF-1 levels and binding of AUF1 to their respective promoters would lead to enhanced EMT, which was due to the increased expression of SDF-1, α-SMA, TGF-β1, and IL-6 [70]. Here, we confirmed that PGRN promoted the malignant biological behavior of PTC cells by activating the JAK2-STAT3/4 signaling pathway. Kurnellas et al. found that plasma PGRN and cerebrospinal fluid (CSF) PGRN were increased by using latozinemab in the first-in-human phase 1 clinical trial, which were beneficial for the treatment of *GRN* mutation causing frontotemporal dementia (FTD-*GRN*) and other neurodegenerative diseases [71]. And the findings in study suggested that developing antagonists targeting PGRN might be a potentially effective therapeutic strategy for PTC. What’s more, since PGRN was associated with proliferation, invasion, and metastasis, it might be helpful for patients to choose the surgical methods, decide whether to perform preventive central lymph node dissection and the extent of lymph node dissection. The limitations existing in the study are the lack of additional in vivo evidence. The study of tumor-related genes only partially explained the molecular mechanism of tumor occurrence and development. The evolution from a normal cell to a cancer cell and then to form a tumor and metastasize, in addition to the genetic variation, it is also closely related to the functions of its microenvironment, including the surrounding mesenchymal cells, immune cells and related endothelial cells. These questions will be the object of our further study.

## Conclusion

In conclusion, the results showed that PGRN was highly expressed in PTC, and the expression level of PGRN was associated with lymph node metastasis in patients with PTC. We also obtained that PGRN promoted PTC cell proliferation, G0/G1 phase to S phase transition, metastasis, and inhibited PTC cell apoptosis by JAK2-STAT3/4 signaling pathway. The findings indicated that developing antagonists targeting PGRN might be a potentially effective therapeutic strategy for PTC. Next, we would like to provide more evidence in vivo and make more contribution to the effect of PGRN on PTC.

## Data Availability

The authors declare that all data used or analyzed during the current study are available on reasonable request.

## Abbreviations

ATC: Anaplastic thyroid carcinoma
CSF: Cerebrospinal fluid
CTSD: Cathepsin
EDTA: Ethylenediaminetetraacetic acid
EDU: 5-ethynyl-2’-deoxyuridine
EMT: Epithelial–mesenchymal transition
ETE: Extrathyroidal extension
FTD: Frontotemporal dementia
GSEA: Gene set enrichment analysis
JAK: Janus kinase
KEGG: Kyoto encyclopedia of genes and genomes
MMP2: Matrix metalloproteinase 2
PBS: Phosphate buffered saline
PGRN: Progranulin
PSAP: Prosaposin
PTC: Papillary thyroid carcinoma
PTMC: Papillary thyroid microcarcinoma
qRT-PCR: Quantitative reverse-transcription polymerase chain reaction
SLPI: Secretory leucocyte protease inhibitor
SORT1: Sortilin1
STAT: Signal transducer and activator of transcription
TCGA: The cancer genome atlas
